# Rapid Genomic Characterization of the Genus *Vitis*

**DOI:** 10.1371/journal.pone.0008219

**Published:** 2010-01-13

**Authors:** Sean Myles, Jer-Ming Chia, Bonnie Hurwitz, Charles Simon, Gan Yuan Zhong, Edward Buckler, Doreen Ware

**Affiliations:** 1 Institute for Genomic Diversity, Cornell University, Ithaca, New York, United States of America; 2 Cold Spring Harbor Laboratory, Cold Spring Harbor, New York, United States of America; 3 New York State Agricultural Experiment Station, Geneva, New York, United States of America; 4 United States Department of Agriculture, Agricultural Research Service, Ithaca, New York, United States of America; University of Umeå, Sweden

## Abstract

Next-generation sequencing technologies promise to dramatically accelerate the use of genetic information for crop improvement by facilitating the genetic mapping of agriculturally important phenotypes. The first step in optimizing the design of genetic mapping studies involves large-scale polymorphism discovery and a subsequent genome-wide assessment of the population structure and pattern of linkage disequilibrium (LD) in the species of interest. In the present study, we provide such an assessment for the grapevine (genus *Vitis*), the world's most economically important fruit crop. Reduced representation libraries (RRLs) from 17 grape DNA samples (10 cultivated *V. vinifera* and 7 wild *Vitis* species) were sequenced with sequencing-by-synthesis technology. We developed heuristic approaches for SNP calling, identified hundreds of thousands of SNPs and validated a subset of these SNPs on a 9K genotyping array. We demonstrate that the 9K SNP array provides sufficient resolution to distinguish among *V. vinifera* cultivars, between *V. vinifera* and wild *Vitis* species, and even among diverse wild *Vitis* species. We show that there is substantial sharing of polymorphism between *V. vinifera* and wild *Vitis* species and find that genetic relationships among *V. vinifera* cultivars agree well with their proposed geographic origins using principal components analysis (PCA). Levels of LD in the domesticated grapevine are low even at short ranges, but LD persists above background levels to 3 kb. While genotyping arrays are useful for assessing population structure and the decay of LD across large numbers of samples, we suggest that whole-genome sequencing will become the genotyping method of choice for genome-wide genetic mapping studies in high-diversity plant species. This study demonstrates that we can move quickly towards genome-wide studies of crop species using next-generation sequencing. Our study sets the stage for future work in other high diversity crop species, and provides a significant enhancement to current genetic resources available to the grapevine genetic community.

## Introduction

The aim of genetic mapping studies is to identify loci that underlie phenotypic variation. Genetic mapping studies are critical for improving crops through marker-assisted breeding and for our understanding of the relationship between genotype and phenotype [1]. Genome wide association (GWA) mapping [2] and genomic selection (GS) [3] are increasingly being adopted for crop improvement and they often require large numbers of genetic markers. One of the main challenges in agricultural genetics is to access and use the tremendous genetic variation present in germplasm collections and in the wild, as crop species are far more diverse than the vertebrate systems used in biomedical research. To do this, approaches for applying next generation sequencing technology to non-model systems need to be developed [4].

The first step towards GWA and GS is to discover large numbers of genetic markers, generally single nucleotide polymorphisms (SNPs), across the genome. This initial step of large-scale SNP discovery is already underway in several organisms. For example, in humans the International HapMap Project currently boasts over 3 million SNPs (http://www.hapmap.org/), and similar projects are in progress for *Arabidopsis thaliana* (http://walnut.usc.edu/2010), rice (http://irfgc.irri.org) and maize (http://www.panzea.org/). While previous SNP discovery initiatives relied on laborious and relatively expensive sequencing and genotyping platforms, SNP discovery has become less time consuming and much more cost-effective since the introduction of next-generation sequencing (ABI's SOLiD, Illumina's Genome Analyzer and Roche's 454). SNP discovery using next-generation sequence data is still in its infancy, but several studies have already demonstrated that large numbers of high quality SNPs can be identified in a cost effective manner using next-generation sequence data [5]–[9]. Deep sequence coverage across many samples is generally desired in order to identify high quality SNPs. To achieve an increase in coverage, the portion of the genome that is sequenced can be reduced by constructing reduced representation libraries (RRLs). RRLs are generated by digesting each sample with a common restriction enzyme before sequencing and they have been useful for large-scale SNP discovery in several organisms [8]–[11].

After large-scale SNP discovery, it is crucial to gain an understanding of the pattern of linkage disequilibrium (LD) and population structure in the species of interest. The strategy underlying GWA and GS is to genotype enough markers across the genome so that functional alleles will likely be in LD with at least one of the genotyped markers [12]. Thus, an assessment of the rate of LD decay is essential in estimating the number of SNPs required for GWA and GS studies. For example, it has been shown that 500,000 SNPs provide reasonable power for GWA in humans [13] and that 140,000 SNPs provide reasonable coverage of the 125 Mb *Arabidopsis thaliana* genome [14]. An evaluation of population structure in the species of interest is also crucial: it allows the selection of germplasm for a mapping population that will maximize genetic diversity, and thus the number of QTL that can be detected. Numerous studies have recently used genome-wide SNP data to characterize patterns of population structure in domesticated species as a starting point for GWA and GS [15]–[17].

Here we describe the initial steps we have taken towards genome-wide genetic mapping studies in the world's most economically important fruit crop, the grapevine (genus *Vitis*). The grapevine is a long-lived woody perennial consisting of dozens of species whose natural habitat spans the northern hemisphere [18]. The cultivated grapevine, *V. vinifera*, represents one of the earliest domesticated fruits [19] and there are currently ∼19 million acres under vine (http://faostat.fao.org/). Previous characterizations of the genetic structure of the grapevine have been restricted to small numbers of microsatellites [20] or a few hundred informative SNPs [21]–[23]. The grapevine is diploid, has a relatively small genome size (475 Mb) and was recently sequenced by two independent groups [24], [25]. Genetic mapping in the grapevine has relied almost exclusively on linkage mapping, which is time-consuming because of the grapevine's long generation time (generally 3 years). These considerations make the grapevine an ideal candidate for assessing the utility of next-generation sequencing and genotyping arrays in characterizing genome-wide patterns of genetic diversity of a high-diversity, domesticated plant species in order to move rapidly towards GWA studies.

Here we describe a simple and rapid procedure for identifying hundreds of thousands of SNPs from 11 *V. vinifera* cultivars and 6 wild *Vitis* species. From these data, we assess patterns of segregation within and between *V. vinifera* and wild *Vitis* species and provide the most comprehensive analysis of LD decay in *V. vinifera* to date. We also describe the design of a SNP genotyping array for the grapevine that assays 8898 SNPs (the Vitis9KSNP array). We show that the Vitis9KSNP array provides sufficient high-quality genotypes to successfully capture the genetic structure within and between the *V. vinifera* cultivars and wild *Vitis* species. Our analyses suggest that the use of SNP arrays for WGA studies will be inadequate for high-diversity plant species in which LD decays rapidly, as in the grapevine. We suggest a stronger focus on experimental design in the anticipation that future mapping populations will be cost-effectively whole-genome sequenced in the near future.

## Results

**Figure 1:**
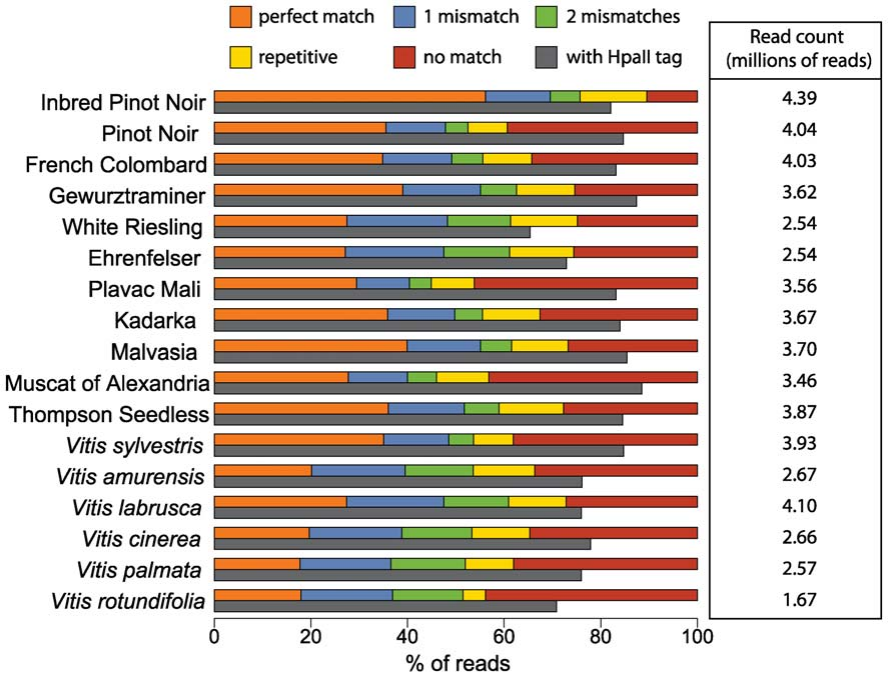
Alignment results of Illumina GA reads to the grapevine reference genome. The total number of reads generated for each sample is found in the box to the right. The upper bars in the barplot indicate the proportion of reads belonging to each of the categories in the legend. Reads aligning with 0 to 2 mismatches were included for SNP discovery. Reads mapped repetitively and reads with no match were discarded. The lower bars (dark grey) show the proportion of reads beginning with the HpaII tag. The wild *Vitis* samples are shown in italics.

We generated reduced representation libraries (RRLs) from 17 grapevine DNA samples (10 cultivated *V. vinifera* varieties, 6 wild *Vitis* species and the reference genome (inbred Pinot Noir) – see Table S1 for details on samples) by digesting each sample with the restriction enzyme *HpaII*, which has proved useful in the generation of RRLs by others [26], [27]. The generation of RRLs permits high-coverage sequencing of a small, similar fraction of the genome across samples. Each RRL was sequenced on a single lane of Illumina's Genome Analyzer to produce 57.3 million 36-bp reads (2.6 Gb of DNA sequence). We trimmed off the last 4 bases of each read and aligned the 32 bp reads to the reference genome using ELAND (Illumina Inc). In total, 68% of the reads successfully mapped to the reference genome: 57% mapped uniquely, 11% mapped to multiple locations (repetitive) and 32% provided no alignment (no match). Figure 1 provides a summary of the alignment results and the proportion of reads carrying the *HpaII* sequence tag across the 17 samples.

**Figure 2:**
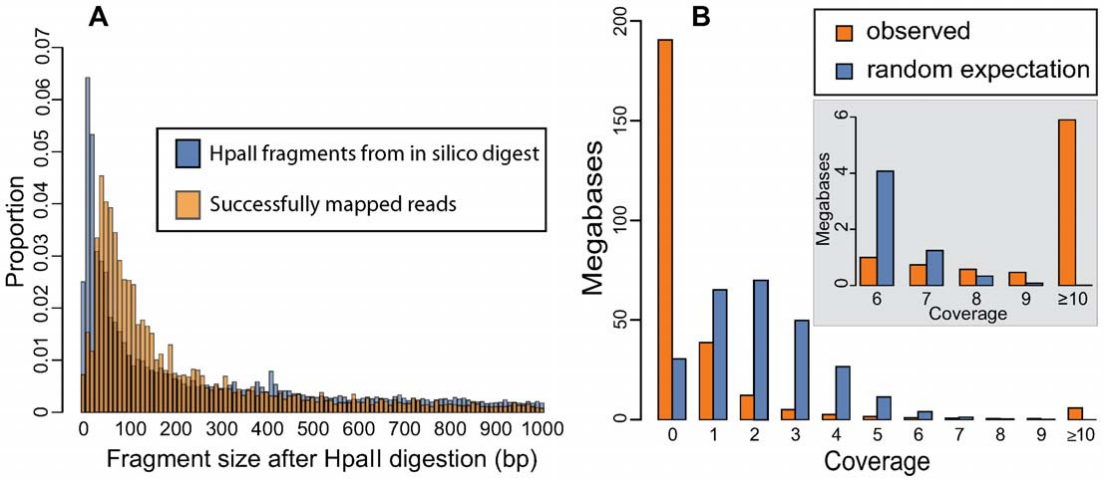
HpaII digestion results in an enrichment of genomic regions with high read coverage. Panel A presents two overlapping fragment size distributions. The size distribution of fragments from an *in silico* HpaII digestion are shown in blue and the size distribution of the *in silico* digested fragments to which reads were successfully mapped is shown in orange. Panel B compares the observed amount of the genome sequenced at each level of coverage to the expectation at random. The random expectation was generated assuming that coverage follows a Poisson distribution (see Methods for details). The inset in gray demonstrates that the observed coverage begins to exceed the random expectation at 8x coverage. SNPs were called from positions with ≥10x coverage.

The sequencing was clearly enriched for successfully digested fragments as 81% of the sequence reads began with the *HpaII* sequence tag (CGG). Figure 2 summarizes the extent to which the sequencing of the RRLs resulted in higher than expected coverage of a small fraction of the genome. We observed a strong enrichment of reads mapping to *HpaII* digested fragments between 40 bp and 250 bp (Figure 2A), which is likely the result of PCR and cloning biases in the Illumina system. In addition, we compared the observed coverage to the coverage expected if no enrichment procedure had been performed (Figure 2B). Our enrichment procedure resulted in more bases covered at 0x and ≥8x than expected if no enrichment procedure had been performed (Figure 2B; see Methods for details). Thus, the use of RRLs concentrated the sequencing on a smaller portion of the genome which provided high enough coverage for reliable SNP calling.

**Figure 3:**
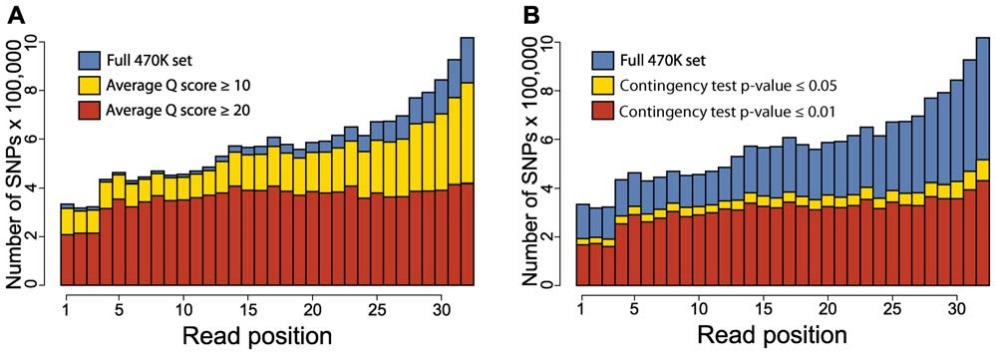
Quality score (Q) and genotypic contingency test thresholds eliminate read position bias during SNP calling. The 470K SNP set is enriched with SNPs identified from the ends of reads. Panel A demonstrates that this read position bias can be eliminated by applying a Q score threshold. Panel B demonstrates that the genotypic contingency test also improves SNP calling.

**Figure 4:**
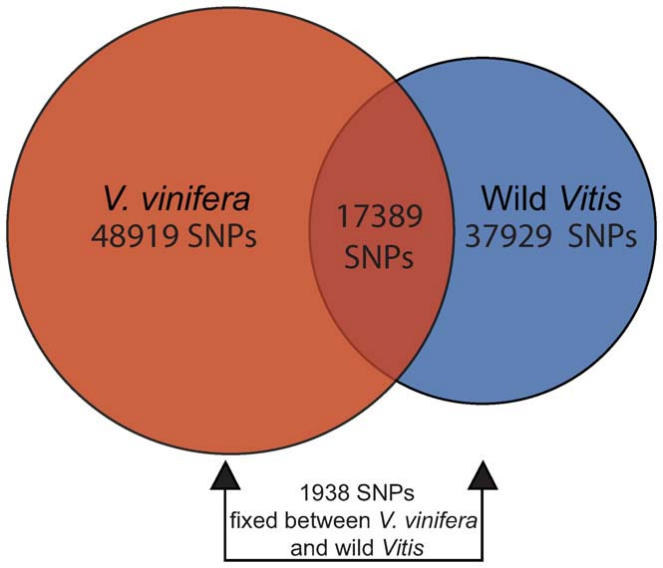
Segregation of SNPs in the 71K SNP set within and between *V. vinifera* and wild *Vitis* species. The proportion of SNPs polymorphic only within *V. vinifera* is 68.5%. The proportion segregating only within wild *Vitis* species is 53.1%. A substantial proportion (24.3%) of SNPs shows evidence of segregation within both *V. vinifera* and the wild *Vitis* species. Only 2.7% of SNPs appeared fixed between *V. vinifera* and wild *Vitis*.

After aligning all of the reads to the reference genome and applying some preliminary filters (see Methods), we identified 469,470 SNPs, which we refer to as our 470K SNP set. Figure 3 demonstrates that SNPs were infrequent within the first 3 bp of reads and enriched towards the ends of reads in our 470K SNP set. The former observation is explained by our library preparation procedure: 81% of reads begin with the CGG-tag and we are therefore unlikely to observe polymorphism within the first 3 bp of reads. The latter observation, however, is consistent with the effects of sequencing error: errors are concentrated towards the ends of reads [5], [8], [28]. This suggests that our 470K SNP set contains false positives which are disproportionately represented at the ends of reads. We found that implementing a strict filter that disregards evidence of polymorphism from the ends of reads resulted in unacceptably high false negative SNP call rates. We therefore investigated several methods that help eliminate the observed read position effect. We found that the two most effective methods were the application of a quality score (Q) score threshold and a threshold on the p-value from a genotypic contingency test. The genotypic contingency test is applied to the read counts at a particular SNP (reference vs. alternative allele across samples) which are represented as a contingency table (see Supplementary Methods S1 for details). Figure 3 demonstrates that these methods are effective in eliminating the bias of SNP discovery towards the ends of reads. Selecting SNPs with average Q scores ≥20 and contingency test p-values ≤0.01 results in a set of 71,397 SNPs which we refer to as the 71K SNP set. The 470K and 71K SNP sets are publicly available at [ftp://brie4.cshl.edu/pub/vitis_plosone_2009_snps/]. SNPs were most often called with coverage from fewer than all 17 accessions. In the 71K SNP set, for example, 95% of SNPs were assayed from ≥7 accessions (see Figure S1). Figure 4 presents the degree of shared polymorphism between the European cultivated *V. vinifera* cultivars and the wild *Vitis* species for the 71K SNP set.

**Figure 5:**
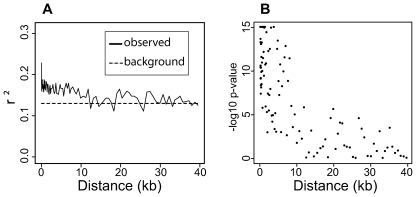
LD decay in the grape. Panel A shows the observed LD decay compared to background LD across 40 kb. LD was calculated as the median r^2^ in bins of 1000 comparisons. The background LD is the median r^2^ from 20,000 comparisons between SNPs on different chromosomes. Panel B shows the –log10 p-values from comparing the distribution of observed r^2^ values within each bin to the distribution of background r^2^ values generated from comparisons between SNPs on different chromosomes using a Mann-Whitney U test (see Methods for details).

To assess patterns of LD decay in *Vitis*, we used a set of simple rules to call genotypes from the Illumina GA sequence data (see Methods). We restricted our analysis to the 10 cultivated *V. vinifera* samples, as each of the wild *Vitis* species was represented by a single sample and there may be significant differences in LD decay between species. Levels of LD are generally low in *V. vinifera* (r^2^<0.2) even at short physical distances (Figure 5A). To determine at what distance LD decays to background levels, we calculated background LD as the degree of LD between SNPs on different chromosomes. We then compared background levels of LD to the observed pattern of LD decay up to 40 kb. Figure 5A demonstrates that while LD is generally low across all distances it remains above background levels to ∼10 kb. To formally test at what distance LD is no longer distinguishable from background LD levels, we compared the observed distribution of r^2^ values in each bin to the 20,000 r^2^ values generated from comparisons of SNPs on different chromosomes using a Mann-Whitney U test (see Methods for details). Figure 5B shows that p-values for these comparisons are consistently highly significant out to ∼10 kb and then begin to decay towards non-significant values.

We designed a custom Infinium SNP genotyping array (Illumina) that assays 8898 SNPs selected from the 470K set by relying on several criteria described in Table S2 and Supplementary Methods S1. We refer to this SNP array as the Vitis9KSNP array. To date, we have genotyped 156 samples with the array and the 94 pairwise comparisons between replicate samples give an average concordance of 99.75%. We compared genotype calls from the Illumina sequence data to genotype calls from the Vitis9KSNP array for the 17 samples (see Methods for details on genotype calling). For 36,904 genotypes called from both datasets, we observe 97.7% concordance. Table 1 summarizes these concordance results by genotype class.

**Table 1:** Concordance of SNP genotype calls.

			Vitis9KSNP array	
		homozygous reference	heterozygous	homozygous alternative
	homozygous reference	24083 (65.26)	285 (0.77)	10 (0.03)
	heterozygous	80 (0.22)	4158 (11.27)	41 (0.11)
	homozygous alternative	29 (0.08)	408 (1.11)	7810 (21.16)

Concordance was assessed for 36,904 SNPs called from both Illumina GA sequence data and the Vitis9KSNP array. Concordance is found along the diagonal and the remaining cells represent different categories of non-concordance. The values inside each cell refer the number of SNPs in that category, followed by the percent value in parentheses. The most common type of non-concordance is found in cases where a SNP is called homozygous from the Illumina data but is called heterozygous from the array data.

**Figure 6:**
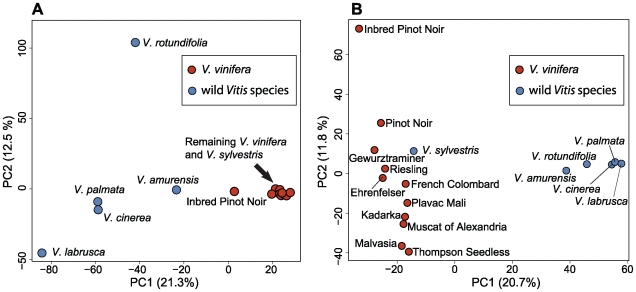
Principal components analysis (PCA) plots from grapevine SNP data. The first two PCs are shown with the proportion of the variance explained by each PC in parentheses. Panel A shows a PCA plot generated from 14,325 SNPs called from the Illumina GA without regard to segregation pattern. Panel B shows a PCA plot from the Vitis9KSNP array data, whose SNPs were chosen purposely to distinguish among *V. vinifera* cultivars.

To investigate patterns of population structure, we performed principal components analysis (PCA) on 14,325 SNPs from the Illumina GA sequence data, which were chosen without regard to the pattern of segregation among the 17 wild and cultivated grapevines (Figure 6A; see Methods for details). In Figure 6A, the first PC, which accounts for 20.7% of the variance, separates wild from *V. vinifera* accessions, while the second PC differentiates among wild species. The exception is *V. sylvestris*, the wild ancestor of the domesticated *V. vinifera*, which clusters with the *V. vinifera* varieties. We also performed PCA on genotype data generated from the Vitis9KSNP array for the same set of 17 samples (Figure 6B). In Figure 6B, the first PC separates wild from *V. vinifera* as in Figure 6A. The second PC, however, differentiates among *V. vinifera* varieties.

## Discussion

WGA and GS studies have generally concentrated on a small number of organisms with established genotyping arrays. With the decreasing costs of DNA sequencing and genotyping, we anticipate that there will be interest in moving rapidly towards GWA and GS studies in organisms for which relatively little genetic data currently exists. Particularly in plants, the Germplasm Repositories of the United States Department of Agriculture currently house over 500,000 different accessions, presenting an enormous amount of genetic diversity to be catalogued and an incredibly large inventory of genetic variation waiting to be discovered and used. In the present study, we provide a framework for rapidly and cost-effectively moving from very few genetic resources, to genome-wide characterization of a species of great economic and cultural interest, the grapevine.

We generated ∼2.6 Gb of DNA sequence using the Illumina Genome Analyser, a substantial proportion (32%) of which did not align successfully to the available genome sequence (Figure 1). Some of these unmatched reads likely come from portions of the genome that are not represented in the current genome, as the genome sequence is not complete. In addition, genetic variation among samples (e.g. highly divergent haplotypes, structural and copy number variation) may result in unaligned reads to the reference genome. For example, the inbred Pinot Noir, which is the identical DNA sample used to generate the reference genome, provided the highest number of successfully aligned reads as expected (Figure 1). Reads from the distantly related wild *Vitis* species matched less often than the cultivated varieties, however several cultivars (e.g. Plavac Mali) showed a low proportion of matches. The variation across samples in the proportion of matches could be due to numerous factors, including variation in exogenous DNA contamination or quality of the sequence data.

Three lines of evidence strongly suggest that our genomic reduction procedure was successful. First, 81% of the sequenced reads begin with the *HpaII* tag (Figure 1). Thus, most of the sequence we obtained came from fragments successfully digested by *HpaII*. Second, there is an excess of reads that map to *HpaII* fragments 40–250 bp in length and a deficit of reads mapping to *HpaII* fragments 0–30 bp in length (Figure 2A). It is known that fragments between 50–250 bp are preferentially amplified on the flow cell of Illumina's GA and this explains our enrichment of reads mapping to fragments in that size range. Finally, Figure 2B demonstrates that the sequencing of RRLs successfully produced an excess of bases with high coverage (≥8x) compared to what is expected without any genomic reduction procedure. Overall, we sequenced 26.4% of the 290 Mb assembled genome to ≥1x coverage and obtained no sequence from ∼73.6% of the assembled genome (i.e. 0x coverage). SNPs were identified only from positions with ≥10x and ≤1000x coverage, which represented only 2.3% of the assembled genome. (A very small portion of the genome was sequenced at >1000x coverage (0.01%)). Although we call SNPs from only 2.3% of the assembled genome, the generation of an equivalent amount of sequence data without an enrichment step would have made large-scale SNP discovery impossible as the required coverage would not have been obtained.

Our genomic reduction procedure and subsequent sequencing enabled the identification of 470K putative SNPs. The excess of evidence for polymorphism at the ends of reads in our 470K SNP set closely resembles the previously described distribution of errors across read positions: the sequencing error rate increases towards the ends of reads [28]. This suggests that the simple SNP calling procedure we implemented to generate the 470K set often does not accurately distinguish between true SNPs and error (Figure 3). Our use of SNP calling criteria based on quality score and the genotypic contingency test (see Methods for details) eliminated this read position bias and resulted in our 71K SNP set. It is also worth noting that indels at the ends of reads may not inhibit alignment and can in some instances be mistaken for SNPs in downstream analyses. SNP calling from short-read sequence data is currently in its infancy, and more sophisticated algorithms exist [6], [29] and will continue to be developed. The fact that the grapevine is highly heterozygous and significantly more genetically diverse than many of the organisms in which SNPs have been called from short-read sequence data [5], [7], [8], makes SNP calling more challenging. In addition, our genome reduction procedure makes it impossible to eliminate the effects of PCR bias as we expect reads to begin and end at the same positions. However, we have demonstrated that a set of simple heuristics can generate a useful data set rapidly and without excessive computational demands. The generation of 71k high-quality SNPs represents a significant enhancement of current genetic resources available to the grape genetics community.

We find relatively few fixed differences (2.7% of SNPs) and a considerable degree of shared polymorphism (24.3% of SNPs) between *V. vinifera* and wild *Vitis* species (Figure 4). The wild *Vitis* species are primarily from North America, but results remain largely the same when *Vitis amurensis*, the only Eurasian wild species in the present study, is excluded from analysis (data not shown). Moreover, this high degree of shared polymorphism is likely an underestimate since polymorphism was often missed due to low read counts. Despite being geographically isolated for more than 20 million years, there is strong evidence of significant degrees of shared polymorphism between North American wild grapevine species and European cultivated grapevines. This observation supports the view that grapevine species have maintained large effective population sizes for millions of years and that, despite having undergone domestication and breeding, *V. vinifera* cultivars still harbor variation that dates back tens of millions of years.

We found that LD decays to background levels at inter-SNP distances of ∼10 kb (Figure 5). Consistent with previous reports [21], [30], levels of LD in *V. vinifera* are low, even at short inter-SNP distances. The median r^2^ for SNPs within 50 bp of each other is only 0.18, for example. This striking observation suggests that the effective population size of the domesticated grapevine is extremely large and historical recombination has fragmented the *V. vinifera* genome into very short haplotype blocks. The rapid breakdown of LD in *V. vinifera*, together with the presence of shared polymorphism between *V. vinifera* and wild *Vitis* species, suggests that grapevine domestication did not involve a severe population bottleneck. Future work assessing levels of diversity and LD decay in *V. sylvestris*, the ancestor of *V. vinifera*, will allow us to quantify more accurately the severity of the domestication bottleneck in the grapevine.

The consequence of the observed rapid LD decay is that genetic mapping in the cultivated grapevine will not follow other organisms' paths towards genome-wide mapping studies. To date, the path towards GWA and GS has begun with genotyping microarrays that carry tag SNPs, SNPs that effectively capture neighboring variants through LD [31]. The grapevine, however, has such low LD that most functional alleles would not be tagged by a genotyped marker from an array-based assay. Thus, we anticipate that whole-genome sequencing will be required for well-powered genome-wide approaches in the grapevine. There are two other reasons why this is a reasonable way to move forward. First, we found that the quality scores from the Vitis9KSNP array are influenced by the number of SNPs present in the probe sequence (Figure S2). This observation suggests that it may be difficult to obtain high-quality genotype data using genotyping microarrays on high-diversity plant species. Second, because the grape is a long-lived perennial that generally produces fruit 3 years after planting, the focus should now be on establishing a mapping population that effectively captures the diversity within the grapevine, paying careful attention to experimental design (e.g. number of replicates, number of environments, etc.). It is likely that by the time sufficient phenotype data is collected from such a mapping population, the sequencing costs will be minimal compared to the costs of establishing and phenotyping the population. Thus, we argue that it is most effective to now concentrate on establishing grapevine mapping populations that will allow for well-powered genetic mapping studies in the future and to exploit the anticipated low future costs of whole-genome sequencing.

To assess the genetic structure of the grapevine, we have designed the Vitis9KSNP array which we are currently using to genotype ∼1200 *V. vinifera* and ∼250 wild *Vitis* species from the USDA's grape germplasm collection. We selected SNPs discovered by Illumina GA sequencing to include on the array based on a number of criteria (Table S2 and Supplementary Methods S1) and observed 97.7% concordance between genotype calls from the Illumina GA data and the genotype calls from the Vitis9KSNP array (Table 1). Table 1 demonstrates that the most common type of error (82% of errors) involves cases in which a SNP is called homozygous from the Illumina GA data but is called heterozygous from the array data. The likely reason for the excess of non-concordant genotypes in these two classes is the presence of polymorphism in *HpaII* sites: an allele at a SNP will not be sequenced if it is linked to an allele that disrupts the *HpaII* site at the start of the sequence. Thus, calling heterozygotes from RRLs is necessarily complicated by the presence of polymorphism within the restriction site, especially in highly heterozygous species like the grapevine. Overall, however, the high concordance rates suggest that the array is providing genotypes that are consistent with the Illumina GA sequence data.

Designing a SNP array to assess the genetic structure of an entire genus is challenging; only a few SNPs that show fixed differences between two species may be necessary to distinguish between them. We intentionally introduced an ascertainment bias during SNP selection for the Vitis9KSNP array and favored SNPs that segregate within the cultivated *V. vinifera*, but also chose a smaller set of SNPs that show fixed differences between each wild species and the *V. vinifera* samples (Table S2). Selecting SNPs for the array strictly based on quality without regard to segregation patterns results in large numbers of SNPs differentiating the wild *Vitis* species. This is apparent in the PCA plot generated from 14,325 SNPs chosen without regard to the pattern of segregation among wild and cultivated grapevines (Figure 5A). For this unbiased SNP set, there is essentially no differentiation among *V. vinifera* until PC4, which accounts for only 7.4% of the variance (Figure S3). When PCA is performed on the same set of samples using the biased set of SNPs from the Vitis9KSNP array, PC1 distinguishes between wild *Vitis* species and *V. vinifera*, and PC2 accounts for 11.8% of the variance and provides clear separation of the *V. vinifera* cultivars (Figure 5B). The exception is the wild species *V. sylvestris*, the known progenitor of *V. vinifera* [18], which is found close to the *V. vinifera* as expected. Inclusion of additional samples that we have genotyped with the array demonstrates that the Vitis9KSNP provides power to distinguish between *V. vinifera*, hybrids and wild species (Figure S4) and even resolves relationships among diverse wild species (Figure S5).

The relative positions of the *V. vinifera* samples along PC2 in Figure 5B suggest that geography may have an influence on the genetic structure of the domesticated grapevine as PC2 reflects the longitude from which these cultivars are believed to have originated. For example, cultivars from Western Europe (Pinot Noir, Gewurztraminer, Riesling, Ehrenfelser and French Colombard) are concentrated at the top of PC2 while cultivars of eastern origin are found at the bottom of PC2 (Plavac Mali from Croatia; Kadarka from Hungary; Muscat of Alexandria from Egypt; Malvasia from Greece and Thompson Seedless from Iran). The *V. sylvestris* sample in Figure 5B is from Tunisia, so its position along PC2 is also consistent with the longitudinal gradient. Only a small number of accessions have been analyzed here and the results from our analyses of Vitis9KSNP array data from the entire USDA grape germplasm collection promises to provide a more in-depth view of the genetic structure of the cultivated grape.

Having assessed the diversity of the grapevine using a whole-genome sequencing approach as well as a genotyping array, it is evident that the choice between using either of these two technologies depends very much on the purpose of the study at hand. The design of a high-quality genotyping array with millions of SNPs for GWA in the grapevine is, arguably, an impossible task because of the difficulties associated with assaying diversity across such a diverse genus. It is our view that next-generation sequencing should and will be primarily utilized for GWA studies in high diversity crop species. On the other hand, customized SNP arrays, such as the Vitis9KSNP in this study, will be valuable for preliminary assessments of germplasm collections and for breeders to verify their material.

## Methods

### SNP Discovery by Illumina GA Sequencing

Genomic DNA was extracted with DNeasy Plant Mini Kits (Qiagen) from young, lyophilized leaves, cambium tissue or leaf bud tissue. Details about the 17 DNA samples are provided in Table S1. DNA samples were amplified with bacteriophage Phi29 DNA polymerase provided in the Genomiphi whole-genome amplification kit (GE Healthcare). We performed a genome complexity reduction step by fully digesting each sample with the restriction enzyme *HpaII* (recognition sequence = CCGG) to generate reduced representation libraries (RRLs). *HpaII* is a methyl-sensitive enzyme, but the genome amplification step prior to restriction digestion eliminates methylation and *HpaII* therefore behaves as a non-methyl-sensitive enzyme in this case. The standard library preparation for Illumina's 1G Genome Analyzer was then performed for each RRL with one alteration: size selection by gel excision was not performed as our experience suggests that it makes no difference in sequence quantity or quality (Ed Buckler, unpublished data). Each RRL was sequenced on a single lane of the Genome Analyzer with 36 cycles to produce 57.3 million reads. The sequences generated in this study have been submitted into the NCBI short read archive (SRA accession: SRA009211.21). Each 36 bp read was first shortened to 32 bp (a requirement for the alignment tool) and aligned to the grape reference genome [24] using Illumina's ELAND alignment tool. In this manner, we detected 2,271,594 positions in the genome where 2 or more alleles were observed (i.e. putative SNPs).

To obtain a robust set of SNPs from this set of 2,271,594 putative SNPs, we implemented a series of preliminary filters. First, we rejected a putative SNP if the read count for the minor allele(s) was ≤5% of the total read count. This filter aims to distinguish between sequencing error, which should be found at low frequency, and true polymorphism. While this filter likely rejected true low-frequency SNPs in some cases, this is of little concern since we were primarily concerned with identifying intermediate-frequency SNPs. Some putative SNPs were covered by >50,000 reads. Putative SNPs covered by extremely high read counts are more likely to be non-allelic, i.e. the result of paralogy: although a set of reads may align to a single genomic location according to the genome sequence, they in fact are derived from multiple genomic locations that are misrepresented as a single sequence in the currently available genome sequence. To mitigate the paralogy problem, we implemented a second filter whereby putative SNPs were rejected if the total read count was >1000. This second filter also aids computational speed. Third, we implemented an arbitrary read count requirement and rejected SNPs with total read counts <10. Finally, when 3 or 4 alleles were observed, we rejected putative SNPs if the sum of the 3^rd^ and 4^th^ most common alleles was ≥2% of the total read count. We then considered only the two most common alleles as we are only interested in identifying bi-allelic SNPs. The implementation of these preliminary filters resulted in 469,470 SNPs, which we refer to as our 470K SNP set. From the 470K SNP set, we identified a 71,397 high-quality SNPs which we refer to as the 71K SNP set. The 71K SNP set was established by choosing SNPs from the 470K set with average Q scores ≥20 and genotypic contingency test p*-*values ≤0.01. See Supplementary Methods S1 for a detailed explanation of the genotypic contingency test.

### Coverage Analysis

A significant proportion (31.1%) of the grape genome sequence has not been assigned to a chromosome. Another 7.9% of the genome is assigned to chromosomes, but not anchored to a chromosomal location. For our coverage analysis, we considered only the 60.9% of the genome sequence that is assigned and anchored to locations on chromosomes 1 to 19. We refer to this portion of the genome as the “assembled genome”.

A total of 17,326,203 reads (554,438,492 bp) were successfully mapped to the assembled genome. We generated the observed coverage distribution by calculating the coverage for every base in the assembled genome (see Figure 3B). The observed number of bases with no coverage was 234,673,000 bp. Bases can have no coverage because no reads mapped to their location, or because reads cannot be mapped to their location. The latter scenario applies to bases that are unknown (i.e. bases assigned ‘N’ in the genome sequence) and for bases that lie within repetitive regions. We subtracted the number of unknown bases (12,848,811 bp) and the number of bases within repetitive regions (31,282,949 bp) to obtain a more accurate observed number of bases with no coverage (190,541,240 bp). We obtained an estimate of the amount of repetitive sequence in the assembled genome from http://www.genoscope.cns.fr/externe/Download/Projets/Projet_ML/data/annotation/repeats/.

To generate the expectation from sequencing at random without the use of RRLs, we followed the Lander-Waterman model whereby coverage follows a Poisson distribution if sequence is obtained at random from the genome [32]. Similar to the manner in which we obtained the observed number of bases with no coverage above, we calculated the “mappable portion” of the assembled genome by subtracting the number of unknown bases and the number of bases within repetitive regions from the total number of bases in the assembled genome. Thus, we consider 14.6% of the assembled genome essentially unmappable and exclude it from our calculation of the Lander-Waterman coverage distribution. The random coverage distribution was generated from a poisson distribution with λ = 2.14, where λ is the mean coverage. The mean coverage was obtained by dividing the 286,454,112 bp of sequence that maps to the assembled genome by the 258,954,041 mappable bases of the assembled genome.

### Segregation Patterns

We assessed the pattern of segregation within and between *V. vinifera* and wild *Vitis* species using read count data from the 71K SNP set. For this analysis, *V. sylvestris*, the wild progenitor of *V. vinifera*, was included in the *V. vinifera* group. SNPs with ≥1 read carrying the reference allele and ≥1 read carrying the alternative allele within *V. vinifera* were identified as “segregating” or “polymorphic” within *V. vinifera*. The same criteria were applied to the wild *Vitis* species. Fixed differences were identified as SNPs with one allele present exclusively in *V. vinifera* and the other allele present exclusively in the wild *Vitis* species.

### LD Decay

We called genotypes from the raw Illumina GA read data as follows. A genotype was called only if the read count for an individual at that locus was ≥4 reads. Individuals were called homozygous if they carried ≥4 reads for one allele and 0 reads for the other allele. Individuals with ≥4 reads carrying both alleles were called heterozygous. For the analysis of LD decay, only the 10 *V. vinifera* samples were included. D' is an unreliable measure of LD with small sample sizes and we therefore only present r^2^ values. SNPs with ≥2 missing genotypes were excluded. Singleton SNPs were excluded. Using these criteria enabled us to include 16,486 SNPs and provided sufficient resolution to assess LD decay. The genotype calls are likely sufficiently reliable since comparisons between r^2^ values generated from this SNP calling method and from the stricter SNP calling method described below under “*Vitis9KSNP array*” were highly correlated (r^2^ = 0.95, p<1×10^−15^). A table of r^2^ values and their respective inter-SNP distances was sorted by inter-SNP distance. We calculated the median r^2^ in sequential bins of 1000 observations along this table and plotted this value against the mean inter-SNP distance for each bin. Background LD was assessed by calculating 20,000 r^2^ values between pairs of SNPs on different chromosomes. Pairwise LD was calculated using the R package “genetics” which incorporates maximum likelihood phase estimates into the estimation of LD [33].

### Vitis9KSNP Array

We called genotypes from the Illumina sequence data and compared them to genotype calls from the Vitis9KSNP array. We attempted to find a set of rules for calling genotypes from the Illumina sequence data that would provide a sufficient number of SNPs for comparison while minimizing the false positive rate. An individual was called a homozygote at a locus if there were> = 5 reads from that individual mapping to that locus and all these reads carried the same allele at that locus. An individual was considered heterozygous at a SNP if it had ≥8 reads mapping to the position and if it passed the heterozygosity test (see Supplementary Methods S1 for details of the heterozygosity test). Genotypes were considered missing data if they failed these conditions. This genotyping scheme results in 820,612 genotype calls from the Illumina sequence data. Genotypes from the Vitis9KSNP array were called using Illumina's BeadStudio software. Our observations suggest that larger sample sizes improve genotype calling. We therefore included 139 samples in addition to the 17 samples sequenced by the Illumina GA when calling genotypes with BeadStudio. Only high-quality genotype calls are useful in assessing concordance between data sets. We therefore visually inspected genotype cluster plots in Beadstudio and decided on a set of strict quality thresholds (GenCall score≥0.5; GenTrain score≥0.7) for SNP calling. The use of these thresholds resulted in 69,078 genotype calls from the Vitis9KSNP array. The total number of genotypes called from both the Illumina sequence data and the Vitis9KSNP array was 36,904.

### Principal Components Analysis

Principal components analysis (PCA) was performed using the prcomp command in R [34]. Genotypes were called with the BeadStudio software. Genotype calling included 139 samples in addition to the 17 samples sequenced by the Illumina GA. From visually inspecting genotype clusters, we decided on the following genotype quality thresholds for PCA analysis: GenCall score≥0.15 and GenTrain score≥0.5. We excluded SNPs with call rates <0.8 and SNPs that were monomorphic. The application of these criteria resulted in a set of 5840 SNPs used for PCA analysis.

We called genotypes for SNPs in the 71K SNP set from the Illumina sequence data. To do so, we employed the SNP calling criteria described under the heading “LD decay” of the Methods section above. SNPs called in <14 of the 17 samples were excluded. This resulted in a set of 14,325 SNPs for PCA analysis.

## Supporting Information

Methods S1 Supplementary Methods
(0.10 MB PDF)

Figure S1 The distribution of assayed accessions for the 470K and 71K SNP set. In many cases, reads covering a SNP are only obtained from a fraction of the total number of samples sequenced. The histograms partition SNPs by the number of accessions from which reads were obtained.
(0.01 MB PDF)

Figure S2 The effect of neighboring polymorphisms on array-based SNP call quality. Each SNP on the Vitis9KSNP array is queried by a probe sequence that is complementary to the 50 bp of sequence adjacent to each SNP. SNPs within this adjacent probe sequence may reduce probe-sequence hybridization and thus result in poor quality SNP calling. The GenTrain score, along the Y-axis, is a metric of SNP quality assigned to every SNP on the Vitis9KSNP array by Illumina's BeadStudio software. The number of SNPs from the 71K set within each SNPs' probe sequence is shown along the X-axis. The boxplot demonstrates that the GenTrain Score decreases as the number of SNPs present in the probe sequence increases. Thus, obtaining reliable genotype calls using SNP arrays in highly diverse species will be challenging.
(0.16 MB PDF)

Figure S3 Plots of the first 10 PCs generated from 14,325 SNPs chosen without regard to the pattern of segregation among wild and cultivated grapevines. The proportion of the variance explained by each PC is in parentheses above each plot.
(0.08 MB PDF)

Figure S4 A PCA plot of 100 grapevine accessions. The SNP data were generated from the Vitis9KSNP array and only the first 2 PCs are shown. The proportion of the variance explained by each PC is shown in parentheses. The *V. vinifera*, hybrid *Vitis* cultivars and wild *Vitis* species are easily distinguishable along PC1. PC2 distinguishes among *V. vinifera* cultivars. *V. sylvestris*, the ancestor of *V. vinifera*, is found among the *V. vinifera* cultivars as expected.
(0.03 MB PDF)

Figure S5 A PCA plot of 50 wild *Vitis* accessions. The SNP data were generated from the Vitis9KSNP array and only the first 2 PCs are shown. The proportion of the variance explained by each PC is shown in parentheses.
(0.03 MB PDF)

Table S1 Additional information on grape DNA samples used in the present study
(0.20 MB PDF)

Table S2 Criteria used to choose the 8988 SNPs assayed by the Vitis9KSNP custom genotyping array
(0.09 MB PDF)
