# Supplementary material for: Rapid Genomic Characterization of the Genus Vitis
Source: PLoS One. 2010 Jan 13;5(1):e8219. doi: 10.1371/journal.pone.0008219 (PMC2805708; doi:10.1371/journal.pone.0008219)
Supplement: Methods S1 — Supplementary Methods (0.10 MB PDF) [file pone.0008219.s001.pdf]

Text S1: Details of each of the statistical procedures used to select the SNPs for the Vitis9KSNP array

We chose 9630 SNPs to be assayed by an Illumina custom iSelect Infinium genotyping array. We used several filtering criteria to choose these SNPs from the 470K SNPs discovered by Illumina GA sequencing. Table S2 provides an overview of the criteria used to choose the SNPs. Briefly, we preferentially chose SNPs with strong evidence of segregation within the cultivated *V. vinifera* but also included SNPs segregating within the wild *Vitis* species and SNPs that appeared as fixed differences between a single wild *Vitis* species and all other samples. Details of each of the statistical procedures employed are provided below. Of the 9630 SNPs submitted to Illumina, probes were successfully synthesized for 8988 SNPs.

The Genotypic Contingency Test

A genotypic contingency test is used for testing the null hypothesis of independence of rows and columns in a contingency table of read counts, as shown in the following two examples.

SNP 1	Samples					
	A	B	C	D	E	F
**Reference allele**	9	0	8	15	0	5
**Alternative allele**	0	11	0	0	16	6

Contingency Test: $P < 1 \times 10^{-16}$

SNP 2	Samples					
	A	B	C	D	E	F
**Reference allele**	2	0	4	0	1	0
**Alternative allele**	0	1	0	0	0	1

Contingency Test: $P = 0.45$

The P value from the genotypic contingency test provides a useful heuristic for evaluating the evidence of real segregation for a putative SNP. Essentially, the significance value of the contingency test in this case is a measure of the effect that the samples have on the distribution of the reference and alternative alleles. Our reasoning is that a real SNP should show a strong effect. For the example tables above, SNP 1 has a low P value from the contingency test (P < 1

x 10^{-16}) and is therefore likely a real SNP, whereas SNP 2 (contingency test *P* = 0.45) shows no strong evidence of being a real SNP. High *P* values from this test could be due to sequencing error, low coverage and/or alignment to paralogous sequence.

Heterozygosity Test

The "heterozygosity test" in supplemental table 2 refers to a simple binomial test applied to the read counts of the reference and alternative alleles for a single sample.

SNP 1	**Read Count**
**Reference allele**	9
**Alternative allele**	8

Binomial Test: *P* = 0.81

SNP 2	**Read Count**
**Reference allele**	15
**Alternative allele**	4

Binomial Test: *P* = 0.02

A true heterozygote is expected to have approximately the same number of reads for the reference and alternative alleles. The binomial test is used to test if the read counts are distributed according to this 50/50 expectation: large deviations from the 50/50 expectation result in low *P* values. For the example table above, SNP 1 has a high P value from the binomial test (*P* = 0.81) and is therefore likely a true heterozygote, whereas SNP 2 deviates from the 50/50 expectation (*P* = 0.02) and thus shows weaker evidence of true heterozygosity. We considered a *P* value threshold of 0.10: SNPs for which the *P* value of the binomial test < 0.10 failed the heterozygosity test. Failure of the heterozygosity test could be due to sequencing error, PCR bias, or copy number variation.

BLAST Filter

The probes on the Infinium SNP array are complementary to the 50bp of sequence adjacent to a SNP and the assay involves a single base extension reaction. To ensure that each probe we chose hybridizes to only one genomic location and thus queries only one SNP, we BLASTed the

50bp on either side of each of the 470K SNP set. A probe sequence was retained if the BLAST search produced a hit to an alternative genomic location with ≥ 2 mismatches within the first 10bp of the 3' end or > 4 mismatches total. In addition, probe sequences with a SNP from the 470K set within the first 5bp of its 3' end were discarded. Every SNP included on the array passed this BLAST filter.
