# Supplementary figures and images for: Rapid Genomic Characterization of the Genus Vitis
Source: PLoS One. 2010 Jan 13;5(1):e8219. doi: 10.1371/journal.pone.0008219 (PMC2805708; doi:10.1371/journal.pone.0008219)

**470K SNP set**

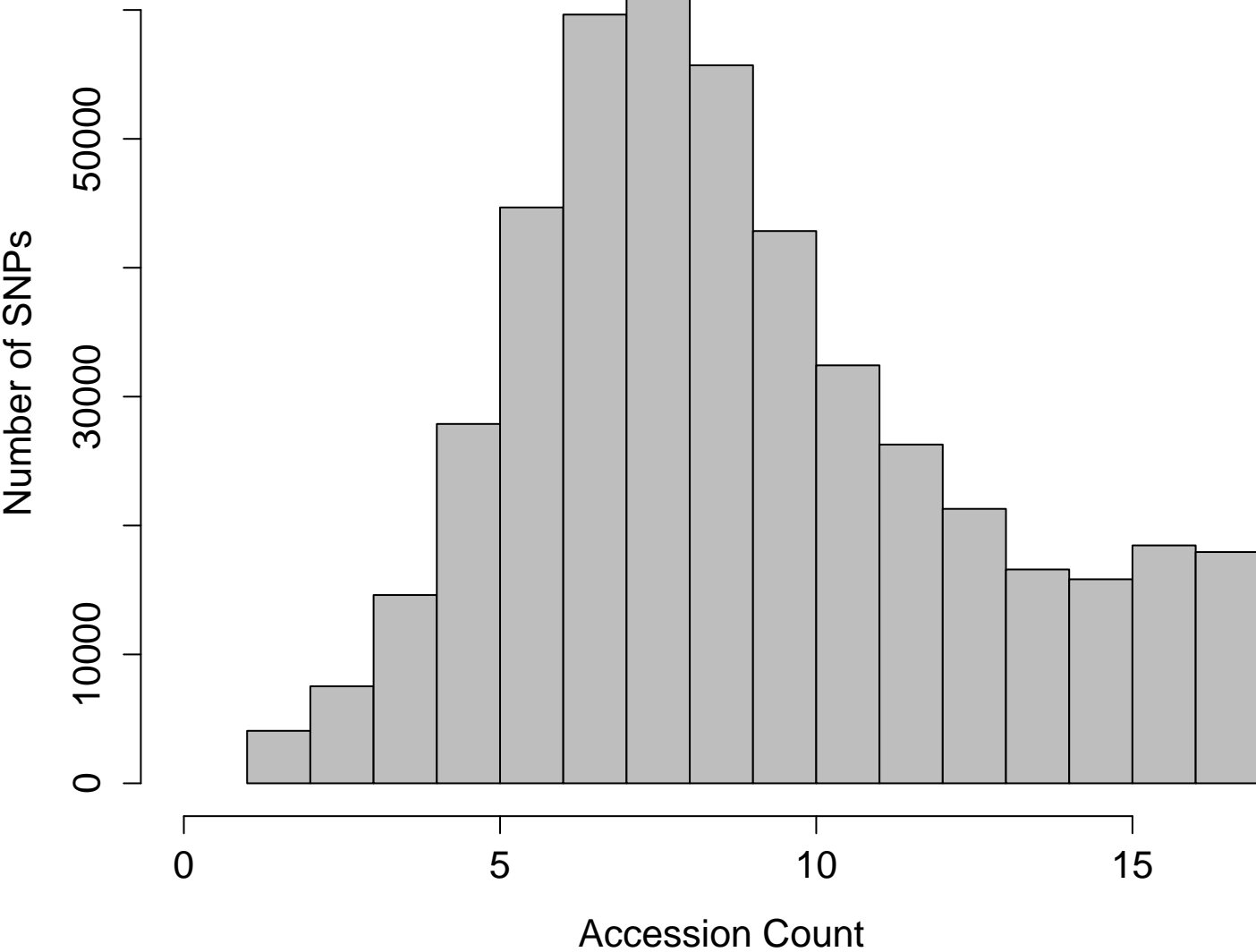

**71K SNP set**

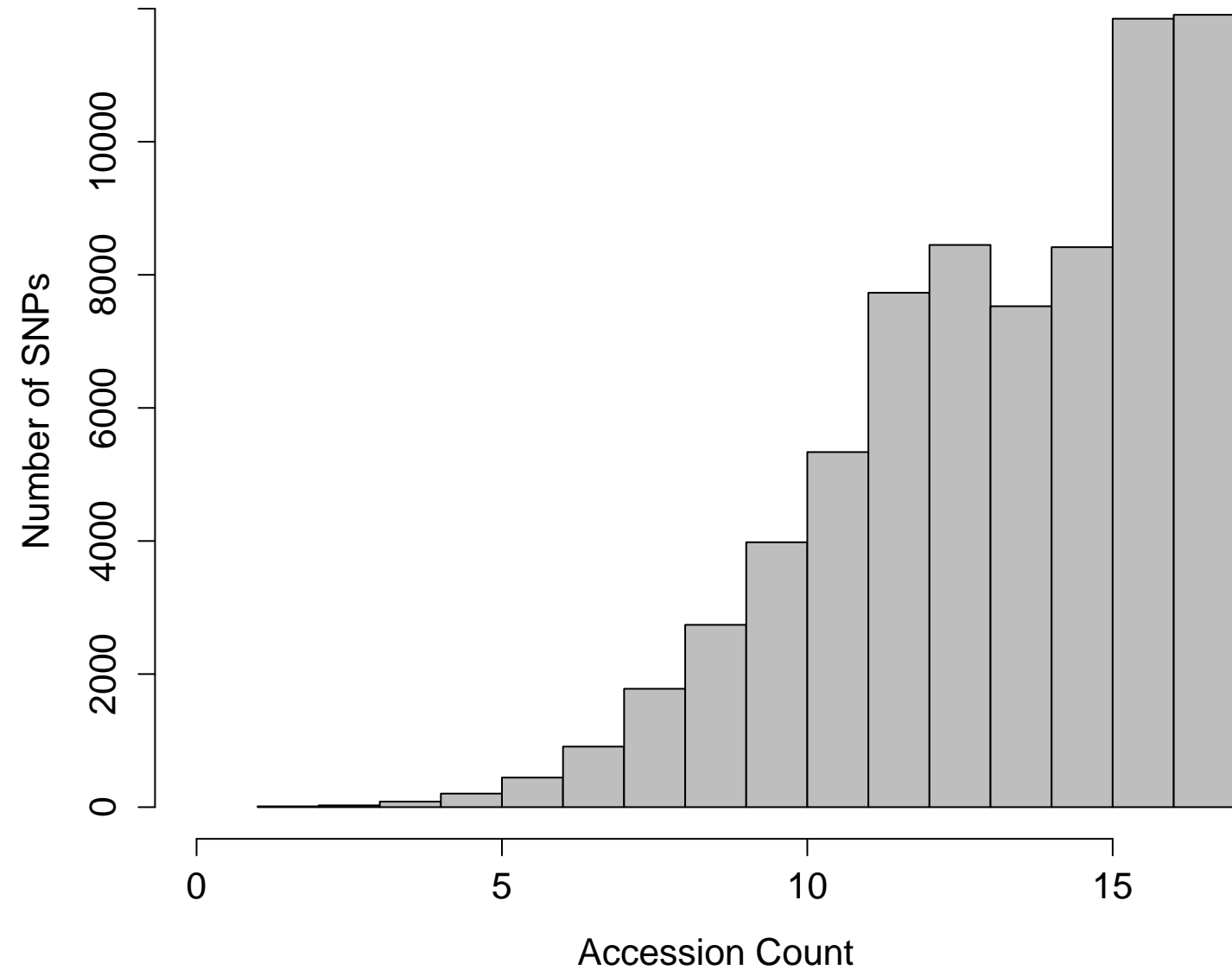

Supplement: Figure S1 — The distribution of assayed accessions for the 470K and 71K SNP set. In many cases, reads covering a SNP are only obtained from a fraction of the total number of samples sequenced. The histograms partition SNPs by the number of accessions from which reads were obtained. (0.01 MB PDF) [file pone.0008219.s002.pdf]

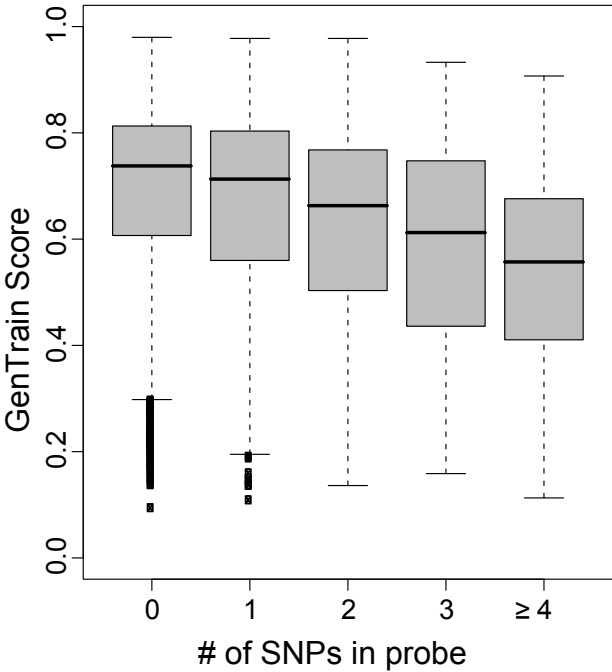

Supplement: Figure S2 — The effect of neighboring polymorphisms on array-based SNP call quality. Each SNP on the Vitis9KSNP array is queried by a probe sequence that is complementary to the 50 bp of sequence adjacent to each SNP. SNPs within this adjacent probe sequence may reduce probe-sequence hybridization and thus result in poor quality SNP calling. The GenTrain score, along the Y-axis, is a metric of SNP quality assigned to every SNP on the Vitis9KSNP array by Illumina's BeadStudio software. The number of SNPs from the 71K set within each SNPs' probe sequence is shown along the X-axis. The boxplot demonstrates that the GenTrain Score decreases as the number of SNPs present in the probe sequence increases. Thus, obtaining reliable genotype calls using SNP arrays in highly diverse species will be challenging. (0.16 MB PDF) [file pone.0008219.s003.pdf]

PC1 (21.3%)

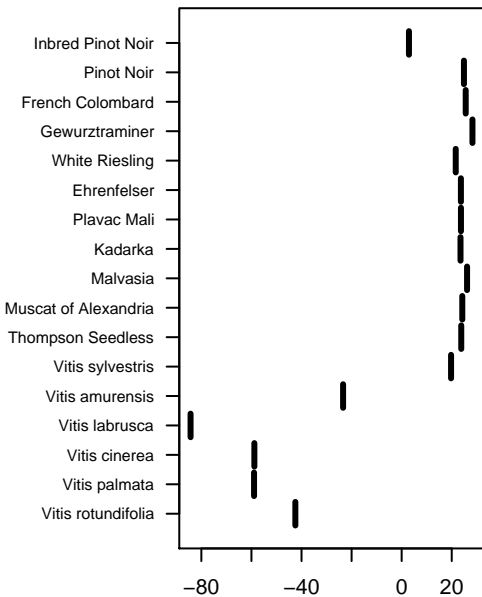

PC2 (12.5%)

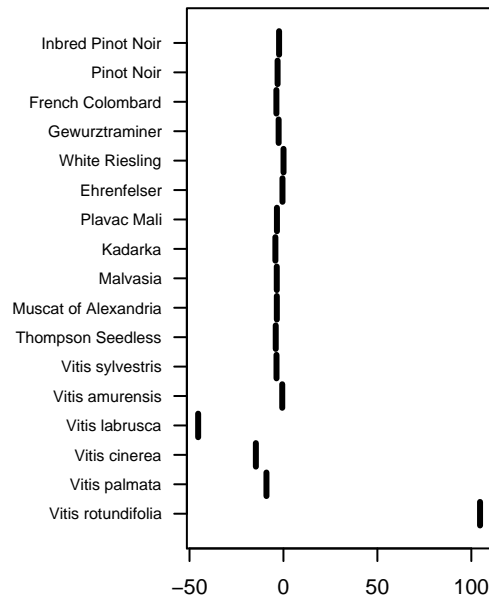

PC3 (8.8%)

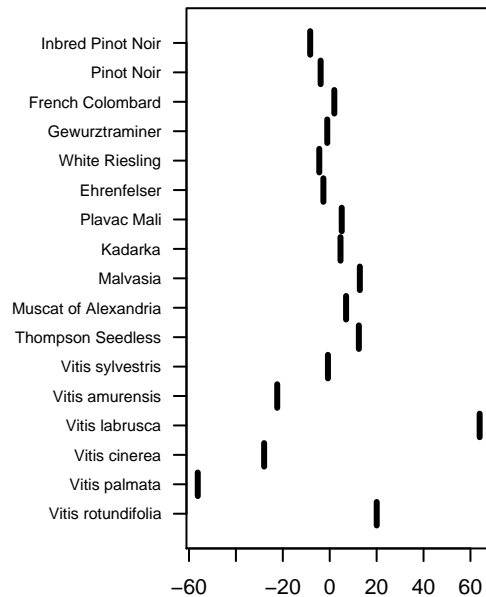

PC4 (7.4%)

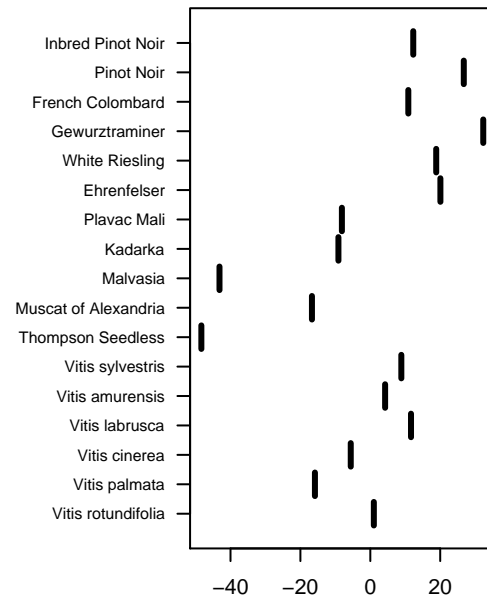

PC5 (6.6%)

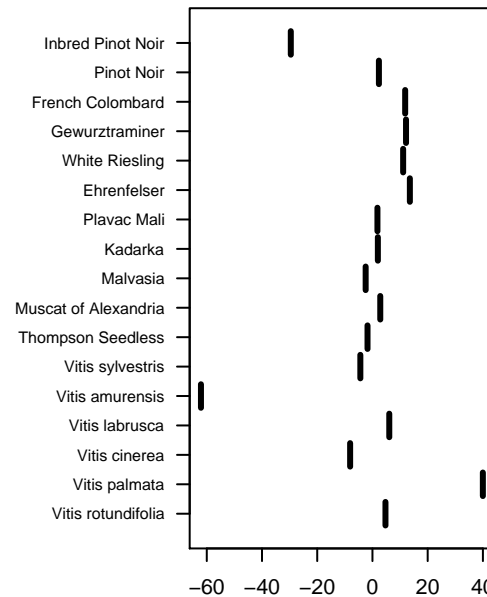

PC6 (5.9%)

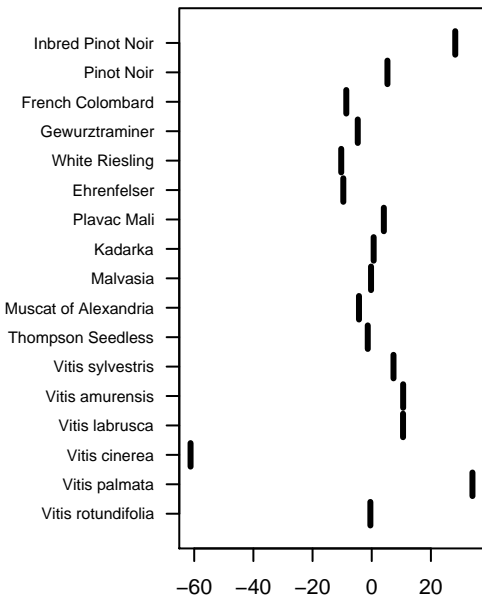

PC7 (5.3%)

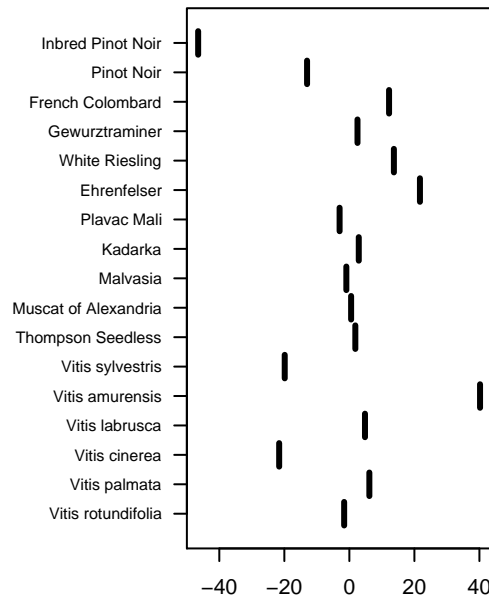

PC8 (4.8%)

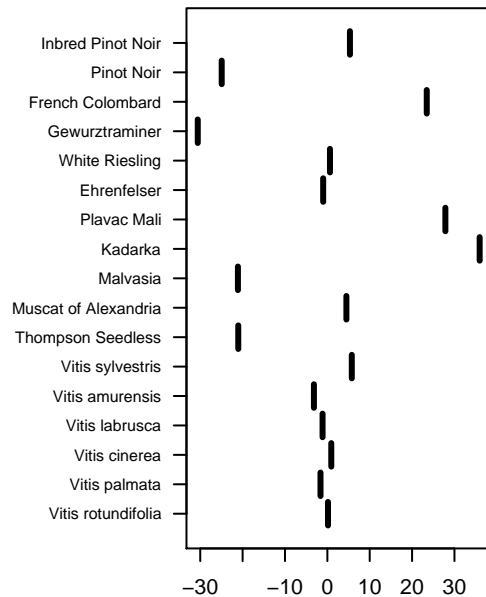

PC9 (4.5%)

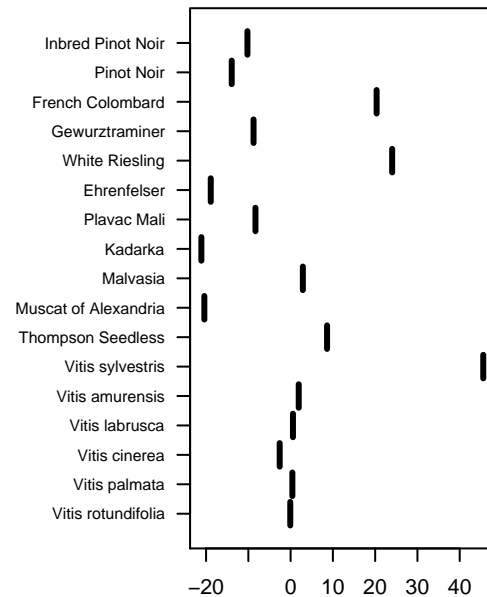

PC10 (4.1%)

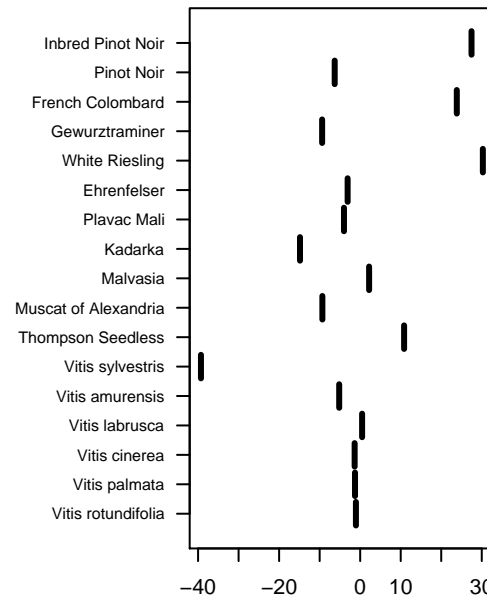

Supplement: Figure S3 — Plots of the first 10 PCs generated from 14,325 SNPs chosen without regard to the pattern of segregation among wild and cultivated grapevines. The proportion of the variance explained by each PC is in parentheses above each plot. (0.08 MB PDF) [file pone.0008219.s004.pdf]

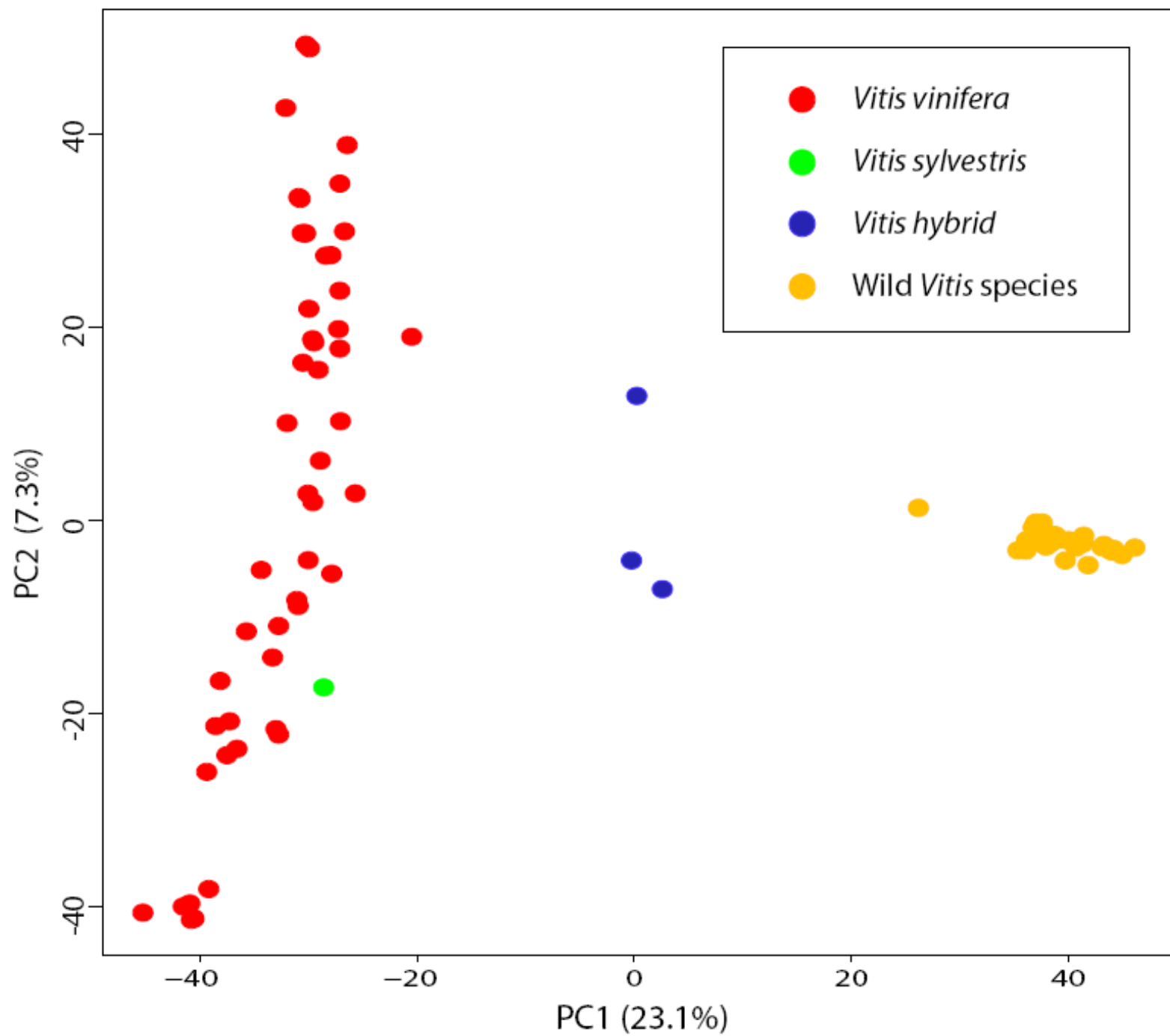

Supplement: Figure S4 — A PCA plot of 100 grapevine accessions. The SNP data were generated from the Vitis9KSNP array and only the first 2 PCs are shown. The proportion of the variance explained by each PC is shown in parentheses. The V. vinifera, hybrid Vitis cultivars and wild Vitis species are easily distinguishable along PC1. PC2 distinguishes among V. vinifera cultivars. V. sylvestris, the ancestor of V. vinifera, is found among the V. vinifera cultivars as expected. (0.03 MB PDF) [file pone.0008219.s005.pdf]

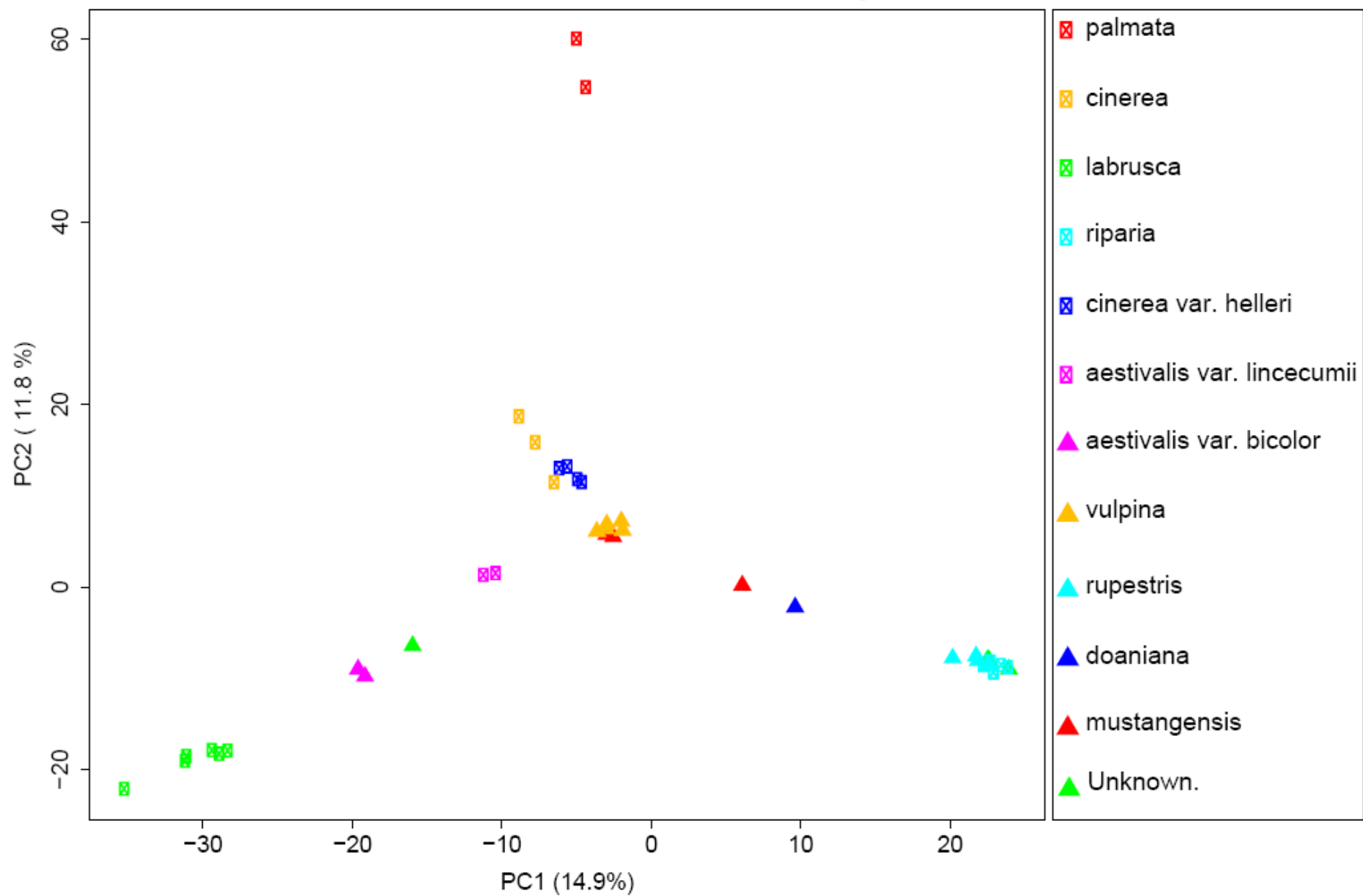

Supplement: Figure S5 — A PCA plot of 50 wild Vitis accessions. The SNP data were generated from the Vitis9KSNP array and only the first 2 PCs are shown. The proportion of the variance explained by each PC is shown in parentheses. (0.03 MB PDF) [file pone.0008219.s006.pdf]
