# Supplementary material for: Rapid Genomic Characterization of the Genus Vitis
Source: PLoS One. 2010 Jan 13;5(1):e8219. doi: 10.1371/journal.pone.0008219 (PMC2805708; doi:10.1371/journal.pone.0008219)
Supplement: Table S1 — Additional information on grape DNA samples used in the present study (0.20 MB PDF) [file pone.0008219.s007.pdf]

Table S1: Additional information on grape DNA samples used in the present study.

**Source of sample**	**Accession identifier**	**Cultivar name**	**Notes**
France	PN40024	Inbred Pinot Noir	Sample provided by Anne Françoise Adam-Blondon INRA, France
Davis, CA USA	DVIT 0915	Pinot Noir	Enter the accession identifier at the following website for additional information: http://www.ars-grin.gov/npgs/acc/acc_queries.html
Davis, CA USA	DVIT 0726	French Colombard	
Davis, CA USA	DVIT 0738	Gewurztraminer	
Geneva, NY USA	PI 588673.02	White Riesling	
Geneva, NY USA	PI 588409	Ehrenfelser	
Davis, CA USA	DVIT 1119	Plavac Mali	
Davis, CA USA	DVIT 0768	Kadarka	
Davis, CA USA	DVIT 0806	Malvasia	
Davis, CA USA	DVIT 0465	Muscat of Alexandria	
Davis, CA USA	DVIT 0535	Thompson Seedless	
Davis, CA USA	DVIT 2426.01	*Vitis sylvestris*	
Geneva, NY USA	PI 588631.03	*Vitis amurensis*	
Geneva, NY USA	PI 483145.01	*Vitis labrusca*	
Geneva, NY USA	PI 588199.01	*Vitis cinerea*	
Geneva, NY USA	PI 588233.01	*Vitis palmata*	
Florida, USA	NA	*Vitis rotundifolia*	Sample provided by Jiang Lu from Florida A&M University Variety name = "Nobel"
