# Supplementary material for: Rapid Genomic Characterization of the Genus Vitis
Source: PLoS One. 2010 Jan 13;5(1):e8219. doi: 10.1371/journal.pone.0008219 (PMC2805708; doi:10.1371/journal.pone.0008219)
Supplement: Table S2 — Criteria used to choose the 8988 SNPs assayed by the Vitis9KSNP custom genotyping array (0.09 MB PDF) [file pone.0008219.s008.pdf]

Table S2: Criteria used to choose the 8988 SNPs assayed by the Vitis9KSNP custom genotyping array. A detailed description of the selection procedures (e.g. genotypic contingency test and heterozygosity test) is provided in the Text S1. The following criteria were applied to the 470K SNP set.

Criterion	# of SNPs	Description
Segregates within *Vitis vinifera* (strict version)	1988	≥ 1 vinifera sample is homozygous (≥ 5 reads) for the reference allele<br>AND<br>≥ 1 vinifera sample is homozygous (≥ 5 reads) for the alternative allele<br>AND<br>≥ 1 vinifera sample passes heterozygosity test (≥ 8 reads)<br>AND<br>Genotypic contingency test *P* value ≤ 0.01 within vinifera<br>AND<br>Average quality score ≥ 20
Segregates within wild *Vitis* species	208	As above but for wild Vitis species
Segregates within *Vitis*	1069	≥ 1 sample is homozygous (≥ 5 reads) for the alternative allele<br>AND<br>≥ 1 sample passes heterozygosity test (≥ 8 reads)<br>AND<br>Genotypic contingency test *P* value ≤ 0.01<br>AND<br>Average quality score ≥ 20
Fixed within a single wild *Vitis* species	1192	One wild Vitis sample is fixed for one allele (≥ 5 reads)<br>and all remaining samples are fixed for the other allele
Segregates within *Vitis vinifera* (lenient version)	3500	[≥ 1 vinifera sample is homozygous (≥ 5 reads) for the alternative allele<br>OR<br>≥ 1 vinifera sample passes heterozygosity test (≥ 8 reads)]<br>AND<br>≥ 1 vinifera sample is homozygous (≥ 5 reads) for the reference allele<br>AND<br>Proportion of vinifera samples that fail the heterozygosity test < 0.2<br>AND<br>Genotypic contingency test *P* value ≤ 0.01<br>AND<br>Average quality score ≥ 20
SNPs within candidate genes	735	SNP falls within a gene from the pre-selected candidate gene list<br>AND<br>Only one allele, reference or alternative, is present in the inbred Pinot<br>AND<br>≥ 10 samples have ≥ 1 read covering this position<br>AND<br>Genotypic contingency test *P* value ≤ 0.01<br>AND<br>Average quality score ≥ 20
Random SNPs	296	
